# Risk of suicide in association with major depressive disorder among patients with dementia: a population-based nested case-control study

**DOI:** 10.47626/1516-4446-2024-3605

**Published:** 2025-01-22

**Authors:** Jiun-Yi Wang, Yi-Ting Hsu, Chih-Yuan Lin, Chien-Hui Liu, Kun-Chia Chang, Chih-Ching Liu

**Affiliations:** 1 Department of Healthcare Administration, College of Medical and Health Science, Asia University, Taichung, Taiwan; 2 Department of Medical Research, China Medical University Hospital, China Medical University, Taichung, Taiwan; 3 Department of Neurology, China Medical University Hospital, Taichung, Taiwan; 4 Neuroscience and Brain Disease Center, China Medical University, Taichung, Taiwan; 5 College of Medicine, China Medical University, Taichung, Taiwan; 6 Institute of Health and Welfare Policy, School of Medicine, National Yang Ming Chiao Tung University, Taipei City, Taiwan; 7 Department of Neurology, Taipei City Hospital, Linsen Chinese Medicine Branch, Taipei City, Taiwan; 8 Institute of Biomedical Informatics, National Yang Ming Chiao Tung University, Taipei City, Taiwan; 9 Division of Emergency Medical Service, New Taipei City Fire Department, New Taipei City, Taiwan; 10 Jianan Psychiatric Center, Ministry of Health and Welfare, Tainan, Taiwan; 11 Department of Psychiatry, National Cheng Kung University Hospital, College of Medicine, National Cheng Kung University, Tainan, Taiwan; 12 Department of Health Care Management, National Taipei University of Nursing and Health Sciences, Taipei City, Taiwan

**Keywords:** Dementia, major depressive disorder, suicide, nested case-control study

## Abstract

**Objective:**

This study aimed to explore the association between major depressive disorder (MDD) and suicide risk in patients with dementia.

**Methods:**

A cohort of 625,218 individuals aged ≥ 40 years with dementia was identified from Taiwan’s National Health Insurance Research Database (NHIRD) between 2007 and 2018. After excluding prevalent cases in 2007, a nested case-control study enrolling 1,256 suicide cases and 5,022 matched controls was conducted. The frequencies of MDD-related outpatient or inpatient visits over a 7-year period preceding the event dates were calculated and analyzed for association using conditional logistic regression.

**Results:**

Dementia comorbid with MDD was associated with increased suicide risk (adjusted OR [AOR]: 2.67), particularly in individuals with ≤ 1.0 MDD episodes per year (AOR: 2.85). A similar association was observed only in individuals aged ≥ 65 years and males, with a pronounced risk of suicide in those experiencing ≤ 1.0 MDD episodes per year (AOR: 3.08 for individuals aged ≥ 65 years; AOR: 3.28 for males). Conversely, the risk increase was evident with > 1.0 MDD episodes per year in those aged < 65 years (AOR: 3.04) and females (AOR: 2.45).

**Conclusion:**

MDD is associated with suicide risk in patients with dementia. The strength of this association possibly varies with age and gender.

## Introduction

Dementia is an increasingly prevalent neurocognitive disorder due to global population aging.^1^ Alongside progressive cognitive decline, nearly 90% of people with dementia experience at least one of psychiatric symptom during the course of their illness.^2^ Depression is the most common such symptom.^3^ It can lead to feelings of worthlessness and guilt, as well as physical limitations such as poor appetite and sleep,^4^ subsequently increasing the burden of health services utilization^5^ and diminishing the overall quality of life in patients with dementia.^3^ At its worst, severe depressive symptoms may even trigger suicidality and elevate the risk of suicide death.^4^ Consequently, mental health conditions, including suicide burden, among people with dementia have become a major concern issue in global public health.^6^

Prior research has identified major depressive disorder (MDD) as a leading cause of death by suicide in the general population.^7^ However, determining whether a similar association exists among people with dementia is crucial for developing interventions aimed at reducing suicide burden for this specific population. Yet, to the best of our knowledge, no study has investigated the association between MDD and the risk of suicide in the dementia population. Only a few studies have included depression cases in their investigation of the risk of suicide in this population, and the findings from these previous studies were inconsistent and limited to individuals in Western countries.^8^,^9^ Among them, one retrospective cohort study with U.S. veterans aged ≥ 60 years with dementia found depression to be associated with increased risk of suicide, with an adjusted odds ratio (AOR) of 2.04 (95%CI 1.45-2.85).^8^ However, in a retrospective cohort study conducted in the U.S. state of Georgia, depression was not identified as a significant predictor of suicide among individuals of all ages with dementia, with an AOR of 1.3 (95%CI 0.7-2.1).^9^ The inconsistent findings from these studies may be attributed to methodological challenges, including a lack of consideration for history and frequency of MDD episodes and a failure to differentiate by age and gender regarding MDD before suicide, leading to inconclusive and potentially biased results.^8^,^9^

Given the aforementioned available studies and methodological limitations in this field, it is imperative to further examine the association between MDD and the risk of suicide. Accordingly, we conducted a nationwide, population-based nested case-control study of patients with dementia in Taiwan to explore whether suicide risk is associated with the history and frequency of MDD episodes across different age groups and genders.

## Methods

### Data sources

Data were obtained from the Taiwan National Health Insurance Research Database (NHIRD) and the national register of deaths, provided by the Health and Welfare Data Science Center. The two nationwide datasets and related official documents were mandatorily collected based on the law.

The NHIRD includes data on outpatient and inpatient medical encounters which are reimbursed by National Health Insurance (NHI). In Taiwan, the NHI program has been implemented since 1995 and covers over 99% of Taiwan citizens.^10^ To confirm the accuracy of claim data in NHIRD, the NHI Administration (NHIA) performs an expert review on a random sample of every 50 to 100 outpatient and inpatient claims quarterly, with false reports of diagnosis receiving severe penalties from the NHIA.^10^ Therefore, information retrieved from the NHIRD are generally regarded as complete and accurate. The NHIRD has been used in many studies on dementia^5^,^11^ and MDD.^12^

The present study was approved by the institutional review board of Jen-Ai Hospital (JAH-110-69).

### Study population

Several steps were taken to identify the dementia cohort for the present study. First, based on a previous study regarding dementia identification using Taiwan’s NHIRD,^5^ we established a cohort comprising 625,218 patients aged ≥ 40 years diagnosed with dementia. These patients had a minimum of three outpatient claim records with ICD-9 Clinical Modification (CM) or ICD-10-CM codes related to dementia diagnosis (Supplementary Table S1) filed from 2007 to 2018, and their initial and final outpatient visits were separated by at least 90 days. Next, considering the heightened risk of suicide in incident dementia cases,^9^ we employed the year 2007 as a screening period to identify patients with incident dementia. This choice was made because patients receiving their initial dementia diagnosis in 2007 might represent existing claim records (prevalent cases) rather than new cases. Hence, these patients were excluded from the cohort. Then, each patient’s personal identification number was linked to the national death registration database in Taiwan to ascertain their mortality status. This linkage process aimed to identify cases of suicide among dementia patients. To specifically identify cases of suicide, individuals who died from non-suicidal causes were excluded from the analysis (n=219,271), and only those who died by suicide within 7 years after their initial clinical diagnosis of dementia were considered cases (n=1,260). This 7-year observational period was chosen based on the median survival time after the first diagnosis of dementia, which is approximately 7 years.^11^

### Nested matched case-control design

Suicide death was defined as the study outcome and retrieved from the national register of deaths in Taiwan (represented by the ICD-10 codes X60-X84, Y10-Y34, and Y87).^13^

**Figure 1:**
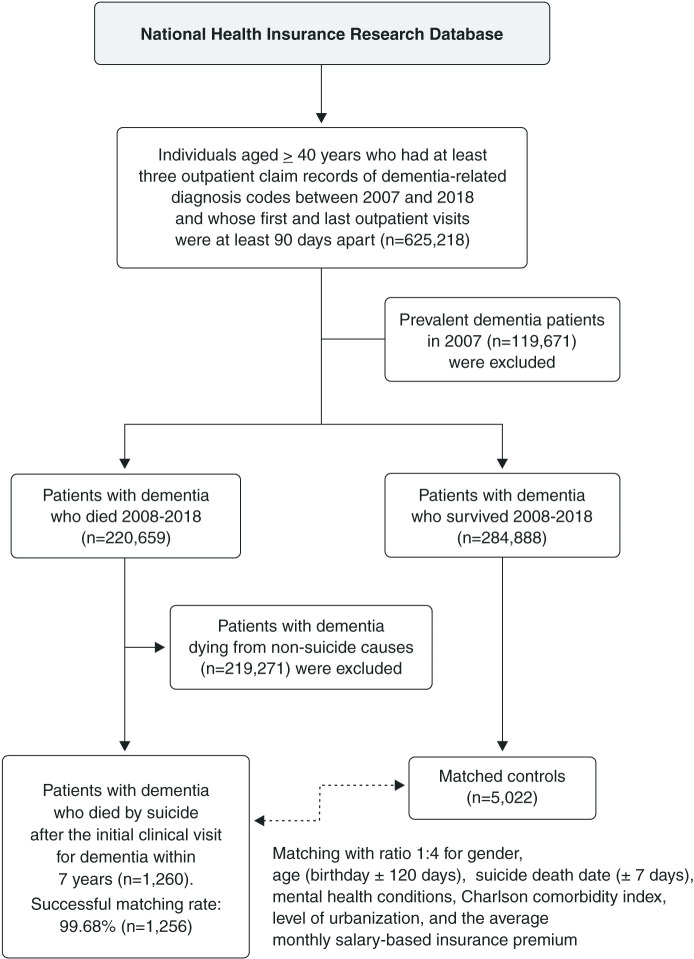
Flow diagram of participant selection for the nested case-control study.

Once the cases of suicide death had been identified, matched controls were selected from the same cohort of dementia patients, which configured the nested case-control study. For each case, those patients with dementia who survived at the suicide death date of cases were chosen as candidates for controls. To establish a comparable control group, we conducted 1:4 propensity score matching with no replacement, based on gender, age (birthday ± 120 days), suicide death date ± 7 days, mental health conditions (anxiety, bipolar disorder, schizophrenia, alcohol-related disorders, and other substance-related disorders), the Charlson comorbidity index (CCI), urbanization level of residence, and the average monthly salary-based insurance premium (categorized into two levels according to the median).^9^,^14^,^15^ The propensity scores of suicide were calculated using logistic regression based on the matching factors, and a greedy matching algorithm was employed to select the closest matches by propensity score,^16^ thereby minimizing selection bias in the control group. Finally, a total of 1,256 cases and 5,022 controls were successfully matched and included in the analysis, with a successful matching rate of 99.68%. A flow diagram of selection of study participants is shown in Figure 1.

### Measurement of major depressive disorder

The exposure of interest was the episode of MDD following the initial clinical visit for dementia. MDD was defined as an inpatient or outpatient visit with an MDD-related diagnosis code (ICD-9-CM codes of 296.2 and 296.3; equivalent ICD-10 codes F32 and F33), due to the well-documented acceptable accuracy of these diagnostic codes.^12^ The frequency of MDD episodes was calculated as the number of MDD episodes per person-year (PY), where the person-years aggregated observational time over a maximum of 7 years from the initial diagnosis of dementia to the date of suicide death. The categorization for frequency of MDD was divided into three groups: 0, ≤ 1, and > 1. Selecting 0 as a cutoff point was logical, given that the median MDD episode frequency was 0 in both the case and control groups throughout the observational period. Setting 1 as the other cutoff point was based on the occurrence of one MDD episode per year in both groups during the observational period. This choice aligns with existing literature indicating that suicide was most likely to occur after having a single MDD episode.^17^

### Identification of covariates

Data on the sociodemographic characteristics were included as control variables, including gender, age at the first clinical visit for dementia, age at suicide death, the average monthly salary-based insurance premium, and the level of urbanization of the place of residence. Both the average insurance premium and the level of urbanization were determined on the basis of records closest in time to, but preceding, day 1 of the observational period. Mental disorders, including anxiety, bipolar disorder, schizophrenia, alcohol-related disorders, and other substance-related disorders, were regarded as potential confounders in the analysis. These mental disorders were identified based on related ICD-9-CM codes (or equivalent ICD-10 codes) appearing at least three times in outpatient records or at least once in inpatient records within 1 year before the observational period. The same algorithm was used to calculate the CCI as a comorbidity score.^18^

### Statistical methods

Difference in categorical variables and continuous variables between the case and control groups were examined using chi-square test and independent *t* test, respectively. To evaluate the association between the history and frequency of MDD and suicide risk, AORs and their 95%CIs were estimated by conditional logistic regression analysis with adjustment for potential confounders. The selection of variables as potential confounders in the model was guided by existing literature and common suicide risk factors, including gender,^9^,^15^ age,^9^,^15^ area of residence,^15^ and mental disorders.^14^,^15^ In addition to these established suicide risk factors, we included the CCI to control for coexisting chronic disorders in the model, as these are also positively associated with suicide.^14^,^19^ Moreover, we adjusted for the average monthly salary-based insurance premium as a proxy for socioeconomic status in the model, considering its known inverse association with suicide.^15^ Additionally, to explore whether age and gender may have an influence on the association between MDD and the risk of suicide, analyses were further stratified by age (< 65 years and ≥ 65 years) and gender during the overall observational period. We also conducted overall, age-specific, and gender-specific analyses to examine the association between the history and frequency of MDD episodes and suicide risk in the up-to-7-year observational period. A p < 0.05 was considered statistically significant. SAS version 9.4 was used to perform all statistical analyses.

## Results

Among the dementia cohort, 1,256 cases and 5,022 matched controls were included. Due to propensity score matching, cases and controls were comparable with regard to gender, age at suicide death, mental health conditions, CCI, level of urbanization, and the average monthly salary-based insurance premium. More than half of the study participants were men, and most were aged 65 years or older. Both groups were more likely to reside in urban areas (48.19 vs. 48.09%) and had a CCI score of ≥ 2 (57.64 vs. 56.47%). The mean monthly salary-based insurance premium for the cases and controls was 19,473.20 ± 16,750.10 and 19,223.90 ± 17,953.30, respectively (Table 1).

The results of conditional logistic regression for the overall, age-specific, and gender-specific associations of the history and frequency of MDD with suicide risk are presented in Tables 2 and 3, respectively. In Table 2, across all participants, cases were significantly more likely to have a history of MDD compared to controls (AOR: 2.67, 95%CI 2.29-3.11). In subgroup analysis, suicide death remained significantly and positively associated with a history of MDD in participants aged < 65 years (AOR: 2.79, 95%CI 2.12-3.69), those aged ≥ 65 years (AOR: 2.50, 95%CI 1.99-3.14), males (AOR: 2.64, 95%CI 2.13-3.27), and females (AOR: 2.37, 95%CI 1.72-3.25). As shown in Table 3, compared to the controls, the case group tended to experience significantly higher frequencies of MDD episodes overall and across age groups and genders. Before and after adjusting for covariates, we observed that participants with any frequency of MDD episodes had a significantly higher risk of suicide death. The AORs were 2.85 (95%CI 2.16-3.74) and 2.61 (95%CI 2.21-3.10) for those who had ≤ 1.0 and > 1.0 MDD episodes per year, respectively, compared to those without MDD episodes. Similar results were also observed in the subgroups age ≥ 65 years and males. While ≤ 1.0 MDD episodes per year were significantly associated with suicide risk in individuals aged < 65 years (AOR: 2.09, 95%CI 1.25-3.49) and females (AOR: 2.07, 95%CI 1.12-3.82), > 1.0 MDD episodes per year corresponded with an even higher suicide risk in these subgroups.

## Discussion

### Main findings

In this large, nested case-control study, we identified a significantly increased risk of suicide among patients with dementia who had a history of MDD within the observational period (i.e., from the diagnosis of dementia to suicide death). The strength of this association was more pronounced among individuals who had ≤ 1.0 MDD episodes per year. However, similar results were only observed in patients aged ≥ 65 years and in males. For patients aged < 65 years and females, the increased risk of suicide was more pronounced in these subgroups when they experienced > 1.0 MDD episodes per year.

### Association between major depressive disorder episodes and risk of suicide

Previous studies on the associations of depression with risk of suicide in patients with dementia were limited and mixed.^8^,^9^ The only two studies on this topic were retrospective cohort designs from the United States,^8^,^9^ one of which found that depression showed no significant effect on suicide in patients with dementia.^9^ Although the other U.S. study found depression to be the most predictive psychiatric disorder for suicide risk among dementia patients (AOR: 2.04, 95%CI 1.45-2.85), the sample was limited to patients covered by the Department of Veterans Affairs aged ≥ 60 years, consisted primarily of males (approximately 98%), and failed to assess the relationship between the status and frequency of MDD and the risk of suicide.^8^ Our study is distinguishable from previous works by its setting in Taiwan and our determination of the exposure to depression, which aims to compare the history and the frequency of MDD with the risk of developing suicide from an overall aspect or by age and gender group. Consistent with the findings from the aforementioned U.S. study,^8^ we demonstrated a significant positive association (AOR 2.85, 95%CI 2.16-3.74) between risk of suicide over the entire observation period and experiencing ≤ 1.0 episodes of MDD per year, compared to patients with dementia who had no MDD episodes at all. Despite a slight attenuation in the strength of this association, suicide remained significantly and positively associated with > 1.0 MDD episodes per year (AOR 2.61, 95%CI 2.21-3.10). The mechanism whereby MDD predisposes dementia patients to suicide may be partly explained by changes in serotoninergic transmission.^20^ These alterations may provoke impulsive and aggressive behavior,^21^ subsequently increasing the risk of lethal suicidal behavior.^22^ Notably, our results show that individuals faced a higher risk of suicide when experiencing a lower frequency of MDD episodes per year rather than a higher frequency, which is in line with a psychological autopsy study conducted in Canada.^17^ These results may be explained by the following factors. First, a lower frequency of MDD episodes may trigger higher levels of impulsive and aggressive behaviors, thereby encouraging suicide.^17^ Second, previous studies found that suicide in patients with depression is associated with no treatment or inadequate prescription or intake of antidepressants.^23^,^24^ Therefore, we suspected that participants with a single episode of MDD may tend to exhibit poor adherence to antidepressant medications, leading to insufficient diagnosis and treatment of depressive disorders, and consequently increasing the risk of suicide.^23^ Third, individuals may hesitate to disclose their mental health issues^25^,^26^ due to the perceived stigma surrounding mental illness,^25^,^26^ limited mental health literacy,^27^ and a lack of familiarity with healthcare professionals.^26^ This reluctance may persist even after receiving an MDD diagnosis, contributing to delays in seeking mental health assistance.^26^,^27^ Consequently, caregivers and healthcare providers may face challenges recognizing physical and emotional changes among patients with comorbid dementia and MDD, hindering timely access to adequate care during hospitalization and after discharge.^5^ This may in turn contribute to an escalation in the severity of MDD, thereby increasing the risk of suicide.^20^ Conversely, participants experiencing > 1.0 MDD episodes per year may be more likely to overcoming the aforementioned barriers to seeking mental health care for MDD than those experiencing fewer MDD episodes, potentially decreasing the risk of suicide.

### Age and gender differences in the association of suicide with major depressive disorder episodes

Our results showed a significantly increased suicide risk associated with any MDD episodes in dementia, but the strength of this association varied across age and gender groups. For those patients aged ≥ 65 years or males, the highest association was found in those experiencing ≤ 1.0 MDD episodes per year. However, the risk of suicide was higher with > 1.0 MDD episodes per year in those aged < 65 years and females. Previous studies have identified age and gender differences in several factors associated with an increased risk of suicide in individuals with MDD, including the severity of depression,^28^ low levels of social support,^29^ impulsivity,^30^ hostility,^31^,^32^ and negative attitudes toward suicide.^33^,^34^ Thus, we suspected that these biological, psychological, and social differences across age and gender may explain our results. For example, negative attitudes toward suicide are more prominent in older adults^33^ and men,^34^ which may stigmatize and create prejudice against their mental illness, thereby discouraging them from accessing mental health services^33^,^35^ and then potentially contributing to increased MDD severity.^28^,^35^ Additionally, increased depressive symptoms can also interfere with help-seeking behavior,^36^ hindering their opportunities to seek assistance for their issues, thereby increasing suicide risk among depressed individuals.^33^ Moreover, a cultural emphasis on “being a man” tends to be associated with higher levels of impulsivity and competitiveness, leading to a preference for more lethal suicide methods.^30^ Men also exhibit higher levels of hostility, which may strengthen the association between depressive mood and suicide.^31^,^32^ Furthermore, older adults have been shown to exhibit higher levels of cognitive control deficits^1^ and neuroticism,^29^ along with lower scores for openness to experience, increased isolation, and decreased social support.^29^ All these conditions may amplify the negative impact of stressful experiences^37^ or contribute to a preference for more deadly suicide methods, consequently increasing the risk of completed suicide.^29^ In contrast, younger adults and females may be more inclined toward self-disclosure^26^ and have higher rates of contact with mental health services.^35^ Therefore, younger adults and females who have a higher frequency of seeking treatment for MDD may tend to represent more severe MDD courses and symptoms, thereby significantly increasing the potential risk of suicide.^20^ However, these explanations were based on the general population; whether they can be used to explain the relationship between MDD and suicide risk among patients with dementia needs further clarification.

The present study has several strengths. First, to the best of our knowledge, this is the first population-based study to investigate the associations of suicide risk with the history and the frequency of MDD episodes among patients with dementia, considering different age groups and genders. With a relatively large sample drawn from the NHIRD, this study provides accessible stratification by age and gender. Second, the case-control design nested within a dementia cohort could minimize potential selection bias.

This study also has several limitations. First, we selected our dementia cohort according to physician-recorded diagnoses reported in medical claims, which might lead to potential disease misclassification. To decrease the likelihood of such misclassification, we managed to solely include dementia patients who had at least three outpatient visits with an ICD code corresponding to a dementia diagnosis, with the first and last visits more than 90 days apart during the study period.^5^ Similarly, exposure to MDD episodes relied on physician-recorded diagnoses in NHIRD medical claims data, which could introduce potential disease misclassification. However, the likelihood of miscoded MDD is expected to be nondifferential and would primarily result in a biased association toward the null value. Besides, limited sociodemographic data in NHIRD led to exclusion of some potential confounders related to MDD and suicide risk, possibly introducing residual confounding bias. Propensity score matching was used to adjust for differences in characteristics, including mental illnesses, between the suicide group and the control group, thereby further minimizing potential confounding. However, residual confounding bias related to mental disorders cannot be eliminated altogether from our study; since our analysis was based on claims data, we only included individuals who sought medical care for mental disorders. Moreover, patients experiencing MDD and suicide may have a higher risk of admission, potentially introducing Berkson bias. However, even if this bias were present, it would likely have minimal impact, as MDD episodes were identified from both outpatient and inpatient claims. As there were few MDD episodes, we did not distinguish between outpatient and inpatient encounters, potentially introducing ambiguity regarding MDD severity. Besides, although including suicidal ideation in the study could provide a more comprehensive risk assessment, we were unable to further examine the association between MDD and suicidal ideation due to the unavailability of relevant claim data. Additionally, the study sample consists of representative participants from Taiwan; therefore, generalizability of our findings to the whole Taiwanese population is to be expected. However, considering the diversity in culture and healthcare systems across countries, willingness to disclose mental health problems^25^,^26^,^38^ and healthcare service hours^26^ are likely to be different. Therefore, caution is warranted when generalizing our findings to other populations. Furthermore, we did not differentiate between dementia types due to data limitations stemming from a lack of information on imaging findings, laboratory data, and symptoms/signs, which constrained a more detailed interpretation of the study results. Lastly, due to the lack of information on pathophysiologic and emotional symptoms data in NHIRD, the study did not allow for an investigation of underlying causal mechanisms. Given the limitations inherent to our study, we recommend that future research adopt a prospective design employing detailed questionnaires to collect information on pathophysiological and emotional symptoms, along with potential confounders like stressful life events. This approach will be helpful for investigating the underlying causal mechanisms to clarify our findings and enhance our understanding of the relationship between MDD and suicide in individuals with dementia. Additionally, employing this approach in future studies will allow for the verification of codes related to depression, thereby minimizing the potential misclassification of MDD and ensuring the accuracy of our results.

In conclusion, a significantly increased risk of suicide was associated with a history of MDD episodes within a period of up to 7 years before suicide death, particularly in those with ≤ 1.0 MDD episode per year. Similar associations were observed in dementia patients aged 65 years or older and males. In contrast, exposure to > 1.0 MDD episodes per year could lead to a higher risk of suicide in patients under 65 years of age and females. The present findings have several important clinical implications. First, they underscore the importance of managing dementia to reduce the risk of MDD. Second, given the frequency of MDD could act as an indicator of vulnerability to suicide events, clinicians should conduct thorough screenings for depressive symptoms in dementia patients to facilitate timely and appropriate treatment. Once MDD has been diagnosed in patients with dementia, clinicians should adopt appropriate management strategies considering variations in age and gender. (For example, our findings highlighted a significantly increased suicide risk associated with any MDD episodes among dementia patients aged 65 years or older, as well as among male patients with dementia – particularly those experiencing ≤ 1.0 MDD episodes annually.) Therefore, it is crucial to conduct a thorough assessment of suicide risk in these patients following their initial MDD diagnosis to ensure appropriate treatment.

## Disclosure

The authors report no conflicts of interest.

## Figures and Tables

**Table 1:** Characteristics of the study participants

Variables†	Case	Control	p-value‡
Total	1,256 (100.00)	5,022 (100.00)	
Gender			0.584
Male	809 (64.41)	3,193 (63.58)	
Female	447 (35.59)	1,829 (36.42)	
Age (years)§			0.095
< 65	426 (33.92)	1,580 (31.46)	
≥ 65	830 (66.08)	3,442 (68.54)	
Mean ± SD	69.73±13.90	70.02±14.19	0.498
Urbanization status||			0.243
Urban	585 (48.19)	2,367 (48.09)	
Satellite city/town	402 (33.11)	1,723 (35.01)	
Rural area	227 (18.70)	832 (16.90)	
Monthly salary-based insurance premium (NTD)||			0.641
≤ Median	627 (49.92)	2,544 (50.66)	
> Median	629 (50.08)	2,478 (49.34)	
Mean ± SD	19,473.20±16,750.10	19,223.90±17,953.30	0.642
Mental disorder||			
Anxiety	559 (44.51)	2,227 (44.34)	0.918
Bipolar disorder	128 (10.19)	487 (9.70)	0.599
Schizophrenia	50 (3.98)	206 (4.10)	0.599
Alcohol-related disorders	106 (8.44)	433 (8.62)	0.836
Other substance-related disorders	69 (5.49)	271 (5.40)	0.892
CCI||			0.755
0	239 (19.03)	982 (19.56)	
1	293 (23.33)	1,204 (23.97)	
≥ 2	724 (57.64)	2,836 (56.47)	
Mean ± SD	2.12 ± 0.05	2.12 ± 0.03	0.902

Data presented as n (%), unless otherwise specified.

CCI = Charlson comorbidity index; NTD = new Taiwan dollar.

† Inconsistency between the numbers of total participants and participants summed for individual variables was due to missing data.

‡ Chi-square test and independent *t*-test for categorical and continuous variables, respectively.

§ Age at suicide death.

|| Before the observational period.

**Table 2:** Association of history of MDD episodes and suicide risk by gender and age

Subgroup/History of MDD episodes	Case	Control		Unadjusted model			Adjusted model		
	n (%)	n (%)	p-value	Crude OR	95%CI	p-value	Adjusted OR† ‡	95%CI	p-value
Overall									
No	880 (70.06)	4,268 (84.99)	< 0.001	Ref.			Ref.		
Yes	376 (29.94)	754 (15.01)		2.45	2.12-2.83	< 0.001	2.67	2.29-3.11	< 0.001
Age									
< 65									
No	281 (59.41)	1,559 (79.83)	< 0.001	Ref.			Ref.		
Yes	192 (40.59)	394 (20.17)		2.56	1.97-3.33	< 0.001	2.79	2.12-3.69	< 0.001
≥ 65									
No	599 (76.50)	2,709 (88.27)	< 0.001	Ref.			Ref.		
Yes	184 (23.50)	360 (11.73)		2.25	1.81-2.80	< 0.001	2.50	1.99-3.14	< 0.001
Gender									
Male									
No	577 (71.32)	2,717 (85.09)	< 0.001	Ref.			Ref.		
Yes	232 (28.68)	476 (14.91)		2.31	1.89-2.83	< 0.001	2.64	2.13-3.27	< 0.001
Female									
No	303 (67.79)	1,551 (84.80)	< 0.001	Ref.			Ref.		
Yes	144 (32.21)	278 (15.20)		2.31	1.71-3.12	< 0.001	2.37	1.72-3.25	< 0.001

MDD = major depressive disorder; OR = odds ratio.

† Estimated from conditional logistic regression model with adjustment for estimate depression, age at initial visit for dementia, age at suicide death, gender, salary-based insurance premium, urbanization, Charlson comorbidity index (CCI), mental disorder (anxiety, bipolar disorder, schizophrenia, alcohol-related disorders, other substance-related disorders).

‡ Age at the initial visit for dementia was excluded from the full model when estimating the age-specific ORs; gender was excluded from the full model when estimating the gender-specific ORs.

**Table 3:** Overall, age-specific, and gender-specific ORs of suicide risk by frequency of MDD episodes

Subgroup/Frequency of MDD episodes†	Case n (%)	Control n (%)	p-value	Unadjusted model			Adjusted model		
				Crude OR	95%CI	p-value	Adjusted OR‡ §	95%CI	p-value
Overall									
0	880 (70.06)	4,268 (84.99)	< 0.001	Ref.			Ref.		
≤ 1	89 (7.09)	166 (3.31)		2.61	1.99-3.41	< 0.001	2.85	2.16-3.74	< 0.001
> 1	287 (22.85)	588 (11.70)		2.40	2.04-2.82	< 0.001	2.61	2.21-3.10	< 0.001
Age									
< 65									
0	281 (59.41)	1,559 (79.83)	< 0.001	Ref.			Ref.		
≤ 1	42 (8.88)	96 (4.92)		1.93	1.17-3.18	0.009	2.09	1.25-3.49	0.005
> 1	150 (31.71)	298 (15.25)		2.76	2.07-3.67	< 0.001	3.04	2.24-4.13	< 0.001
≥ 65									
0	599 (76.50)	2,709 (88.27)	< 0.001	Ref.			Ref.		
≤ 1	47 (6.00)	70 (2.28)		2.81	1.87-4.22	< 0.001	3.08	2.03-4.66	< 0.001
> 1	137 (17.50)	290 (9.45)		2.10	1.64-2.68	< 0.001	2.33	1.81-3.02	< 0.001
Gender									
Male									
0	577 (71.32)	2,717 (85.09)	< 0.001	Ref.			Ref.		
≤ 1	64 (7.91)	104 (3.26)		2.89	2.01-4.14	< 0.001	3.28	2.26-4.76	< 0.001
> 1	168 (20.77)	372 (11.65)		2.15	1.72-2.69	< 0.001	2.47	1.95-3.13	< 0.001
Female									
0	303 (67.79)	1,551 (84.80)	< 0.001	Ref.			Ref.		
≤ 1	25 (5.59)	62 (3.39)		2.13	1.19-3.82	0.011	2.07	1.12-3.82	0.020
> 1	119 (26.62)	216 (11.81)		2.36	1.69-3.30	< 0.001	2.45	1.72-3.49	< 0.001

MDD = major depressive disorder; OR = odds ratio.

† Ratios per person-year (PY) were aggregated over a maximum 7-year observational period before suicide death date.

‡ Estimated from conditional logistic regression model with adjustment for estimate depression, age at initial visit for dementia, age at suicide death, gender, salary-based insurance premium, urbanization, Charlson comorbidity index (CCI), mental disorder (anxiety, bipolar disorder, schizophrenia, alcohol-related disorders, other substance-related disorders).

§ Age at the initial visit for dementia was excluded from the full model when estimating the age-specific ORs; gender was excluded from the full model when estimating the gender-specific ORs.

## Data Availability

The data that support the findings of this study are available from the Health and Welfare Data Science Center (HWDC), Ministry of Health and Welfare (MOHW), but restrictions apply to the availability of these data, which were used under license for the current study, and so are not publicly available. Data are, however, available from the authors upon reasonable request and with permission from HWDC (https://dep.mohw.gov.tw/DOS/cp-5119-59201-113.html).
