# Supplementary material for: Risk of suicide in association with major depressive disorder among patients with dementia: a population-based nested case-control study
Source: Braz J Psychiatry. 2025 Jan 22;47:e20243605. doi: 10.47626/1516-4446-2024-3605 (PMC12679695; doi:10.47626/1516-4446-2024-3605)
Supplement: Supplementary file 1 [file bjp-47-e20243605-suppl1.pdf]

http://doi.org/10.47626/1516-4446-2024-3605

Supplementary Table S1 ICD-9-CM[1] and ICD-10-CM[2] codes for dementia

ICD-9-CM codes	ICD-10-CM codes
290	F03.90, F05, F01.50, F01.51
291	F10.121, F10.14, F10.150, F10.151, F10.159, F10.180, F10.181, F10.182, F10.188, F10.19, F10.221, F10.230, F10.231, F10.232, F10.239, F10.24, F10.250, F10.251, F10.259, F10.26, F10.27, F10.280, F10.281, F10.282, F10.288, F10.29, F10.920, F10.921, F10.929, F10.94, F10.950, F10.951, F10.959, F10.96, F10.97, F10.980, F10.981, F10.982, F10.988, F10.99
294	F02.80, F02.81, F03.90, F03.91, F04, F06.0, F06.1, F06.8
331	G13.2, G13.8, G30.0, G30.1, G30.8, G30.9, G31.01, G31.09, G31.1, G31.83, G31.85, G31.89, G31.9, G91.0, G91.1, G91.2, G91.3, G91.4, G91.8, G91.9, G93.7, G94
046.1	A81.00, A81.01, A81.09

References

1. Centers for Disease Control and Prevention. CDC Archive [Internet]. International Classification of Diseases, Ninth Revision, Clinical Modification (ICD-9-CM). 2021 Nov 3 [cited 2024 Sep 10]. https://archive.cdc.gov/#/details?url=https://www.cdc.gov/nchs/icd/icd9cm.htm

2. Centers for Disease Control and Prevention. National Center for Health Statistics [Internet]. ICD-10-CM. 2024 Jun 7 [cited 2024 Sep 10]. https://www.cdc.gov/nchs/icd/icd-10-cm/index.html#cdc_generic_section_6-special-announcements
